# Informed consent, genomic research and mental health: A integrative review

**DOI:** 10.1177/09697330211066573

**Published:** 2022-02-04

**Authors:** Nina Kilkku, Arja Halkoaho

**Affiliations:** School of Social Services and Health Care, 20615 Tampere University of Applied Sciences, Tampere, Finland; School of Health, 52917 Tampere University of Applied Sciences, Tampere, Finland Corresponding author.

**Keywords:** ethics and mental health, genomic study, informed consent, mental health/psychiatry, nurse

## Abstract

**Background:**

Research on genomics has increased while the biobank activities are becoming more common in different countries. In the mental health field, the questions concerning the potential participants’ vulnerability as well as capacity to give the informed consent can cause reluctancy in recruiting persons with mental health problems, although the knowledge and understanding of mental health problems has remarkable changed, and practice is guided with inclusive approaches, such as recovery approach.

**Aim:**

The aim of this study was to describe the current knowledge of informed consent practices in the context of genomic research on mental health from the nurses’ viewpoint.

**Methods:**

An integrative review was conducted with search from seven international databases. Data consist 14 publications which were analyzed with thematic analysis.

**Ethical considerations:**

Ethical requirements were respected in every phase of the research process.

**Findings:**

Most of the papers were published in USA and between 2000–2010. Eight reports were categorized as discussion papers, four qualitative studies and one quantitative study. The thematic analysis provided information on five themes: complexity with the capacity to consent, mixed emotions towards participation, factors influencing the decision to participate, nurses’ informed consent process competence and variations between consent procedures.

**Discussion:**

In the informed consent practices, there are various aspects which may affect both the willingness to participate in the study and the informed consent process itself. Implications for practice, education, research, and policies are discussed.

**Conclusion:**

There is a need for more updated international research on the topic in the context of different international and national guidelines, legislation, and directives. This study provided a viewpoint to the more collaborative research activities with people with lived experiences also in this field of research following the ideas of recovery approach.

## Introduction

Research on human genes and genomics has increased while the biobank activities are introduced and becoming more common in different countries. In the mental health field, the questions concerning the potential participants’ vulnerability as well as capacity to give the informed consent can cause reluctancy in recruiting persons with mental health problems, although the knowledge and understanding of mental health problems has remarkably changed, and the policies and practice are guided with inclusive approaches, such as recovery approach. This study describes the current knowledge of informed consent practices in the context of genomic research on mental health from the nurses’ viewpoint.

### Background

Globally, almost 30% of people experience a mental health problem during their lifetime (Steel et al. 2014).^1^ In Europe, more than 110 million people are suffering from different mental health conditions, which covers more than 10% of the population.^2^ The high prevalence of mental health distress and disorders highlights the importance of promotion and prevention ^2,3^ as well as the importance to develop community-based services.^4^ However, mental health problems still carry a strong negative stigma. Especially the personal and perceived stigma has remarkable negative effects on everyday life, help-seeking behavior ^5–7^ and research participation.^8^

Research on medical and biomedical topics is regulated by national legislations and international guidelines. The need for these guidelines is based on past inhuman experiments such as those by Nazi doctors during the Second World War.^9^ The incidents led to the establishment of several international regulations, such as Nuremberg code 1947, Declaration of Helsinki 1964 and the Belmont report 1979. Declaration of Helsinki detailed the ethical principles for medical research, for example, informed consent and risk benefit ratio.^10^ Informed consent is based on voluntary choice and special attention must be paid to vulnerable groups. Respect on person’s rights requires evaluation of competence and disclosure of relevant information as a basis of decision-making, enabling the potential participant to agree or to decline.^11^ However, while vulnerability might be considered as an incompetence to consent, the opinion of the people consider as vulnerable must be respected.^12^

People with diagnosis of a mental health problem are often considered as a vulnerable group in research, although the concept itself is vague and used in a variety of ways in different human ethics research guidelines.^13^ Vulnerability can be described in various ways; as a capacity-based vulnerability with impaired capacity to give informed consent; or as a power-based vulnerability that is based on the power differences between the investigator and the potential participant, making the potential participant susceptible to coercion.^14^ Vulnerability is also frequently used to describe the whole group as vulnerable without a more precise view on participants’ unique characteristics. This point of view has its background in history, but in order to avoid an overprotective approach and enable research in the field of mental health, it should not be used as an automatically excluding term.^15^

The viewpoint of inclusion, also in research, follows the recovery approach which is the guiding principle in mental health practice, policies, and guidelines in several countries today.^16^ The recovery approach itself is not new, its roots can already be seen, for example, in the 18^th^ century,^17^ later in consumer and civil rights movements^18^ and as a counter movement challenging the diagnosis-driven biomedical model of mental health care.^17^ The recovery approach emphasizes the person’s self-determination and participation, despite the challenges with mental health, and it is based on positive concepts such as hope and empowerment^19^ and has much in common with person-centredness,^17,20,21^ social inclusion,^22^ and furthermore the human rights.^16^ Despite the long historical roots there is a need for change in attitudes and in behavior for recovery approach become true in practice.^23^

Professionals may be reluctant to recruit persons with mental health disorders in research, especially research concerning genetic risk of mental health problems,^24^ even though genetic counseling may have several positive outcomes, such as lower levels of shame or guilt, deeper understanding of their condition and sense of empowerment^25^ also for individuals with serious mental health problems. One reason for this reluctance can be the lack of knowledge as integration of new knowledge into curricula is slow.^26^ There are no uniform competency requirements for nursing education and related continuing education.^27^ However, 10 genetic core competencies for all health professionals, including nurses, have been introduced.^28^ These include a set of knowledge, skills and attitudes, for example, basic human genetics terminology, family genetic history assessment and ethical, legal, and social implications related to genomics. Although not all nurses do actual genetic counseling, they have an important role as advocates in supporting patients’ autonomy, privacy, and confidentiality in decision-making and in informed consent processes.^28^

Biobanks extensively contribute to research projects by storing large amounts of biological information and data on, for example, schizophrenia-associated studies.^29^ With the increase of genomic testing and studies on genomics, the role of nurses becomes even more important in informed consent procedures.^30^ Biobank consent is often requested in connection with treatment measures. In these cases, the role of the nurse as the patient’s advocate is emphasized. The nurse is often working more closely than other professionals with the patient, and it is easy for patients to ask them for more information.^36^ This provides an opportunity to discuss with the patient and enables informed consent.^37^ Different consents such as broad, dynamic, and electronic are used. Broad consent is used with biobank samples, meaning that no exact information is given on what studies the sample will be used.^31^ Problems may occur if the donor wants to know the findings and, especially, if the findings may also affect the lives of their family members, for example, in the case of a hereditary disease.^32^ In addition to broad consent, the so-called dynamic consent has been used. Dynamic consent has been built to be used in web-based interfaces so that potential biobank donors can clarify their choices regarding sample distribution and use. Dynamic consent could also provide a specific portal through which biobank donors were able to track which studies are utilizing their biospecimens and health data. This not only facilitates potential participants’ consent process but also offers an opportunity for two-way ongoing communicating between researchers and participants. It is notable that dynamic consent is not a specific consent to the research.^34,35^ In several countries, electronic consent has become more common, enabling the potential donor to consider and decide on their participation at home. The importance of a written information is emphasized.^33^

As the research of genomics and genes has expanded,^38^ it is time to investigate the current information on informed consent practices in the context of genomic studies on mental health. The purpose of this study was to provide information to the large education program developed for health care professionals on genomics and genetic studies. The aim of this study was to describe the current knowledge of informed consent practices in the context of genomic research on mental health from the nurses’ viewpoint. In this study the term genomic study covers the studies on human genome in the described context.^38^

## Methods

This integrative literature review was conducted by adapting the five stages of integrative reviews introduced by Whittemore and Knafl.^39^ The first phase, the problem identification, was conducted to clarify the purpose of the review with previous knowledge, to justify the rationale of the study^40^ and to define the search terms according to the aim of the study. This phase was followed by the literature search phase.^39^ The search strategy was developed with the support of a librarian to ascertain the comprehensiveness of the systematic search. Seven international databases were searched without time limits to cover the current knowledge of the topic as widely as possible, using the search strings (genom* OR “bio bank” OR “Biological Specimen Banks” OR “Biological Substance Bank” or biological bank or genetic*) AND (mental* or psychiatric* or psychologic*) AND (ethic* or informed consent or moral or value*) AND (nurse* or nursing).

**Figure 1.**
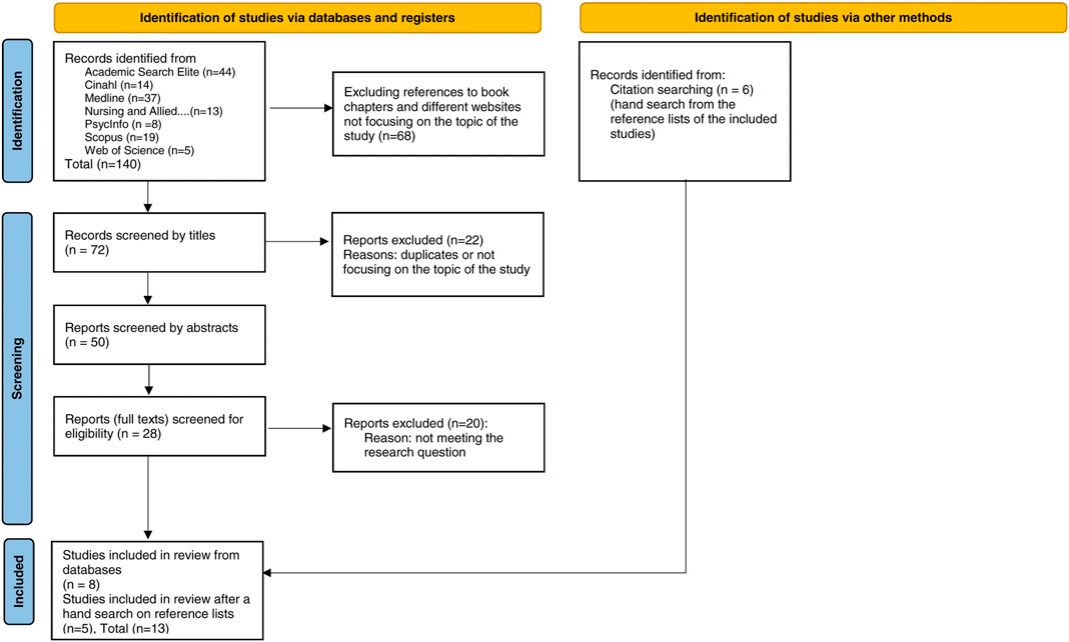
Adapted from: Page MJ, et al. 2021.

The literature search provided 140 reports and after the exclusion process by titles, abstracts and removal of duplicates, the data consisted of eight reports. This exclusion process is described as an adaptation of PRISMA phases.^41^ in the Figure 1. Besides the database search, a hand-search was conducted with the references listed in the chosen reports.^42,43,44^ This revealed four more reports, which were all checked with criteria, and included in the data of 13 reports. The report by Hem et al. (2007)^45^ did not match with the criteria “genom” or “genetic,” but as it was referred to in other reports included in the data, the paper was evaluated as eligible and included (total *n*=13).

The data evaluation phase^39^ revealed the type of the reports, countries of origin and publication year. This categorizing is presented in Table 1. There were altogether eight reports which were categorized as discussion papers, four qualitative studies and one quantitative study. The distinction between the categories of discussion papers or qualitative reports was not obvious in all reports as some used case examples or described the studies as a context or starting point for discussion. However, if no clear qualitative research setting was described as primary, these were included in discussion reports. The country of origin was categorized according to the contact details of the primary author.

**Table 1.** Reports included and categorized (in chronological order).

Author(s)	Year	Country	Category
Williams, J.K. and Schutte, D.L.	2000	USA	Qualitative research
Knoppers, B.M., Avard, D., Cardinal, G., and Glass, K.C	2002	Canada	Discussion paper
Petska, E.	2003	USA	Discussion paper
DeCamp, M. and Sugarman, J.	2004	USA	Discussion paper
Cedar, SH.	2006	United Kingdom	Discussion paper
Hem, M. H., Heggen, K., and Ruyter, K. W.	2007	Norway	Discussion paper
Petska, E. L., Hale, A. M., Johnson, B.L. Lee, J. L., and Poppe, K.A.	2007	USA	Discussion paper
Prows, C.A. and Saldana, S. *N*.	2009	USA	Discussion paper
Bevan, J.L., Senn-Reeves, J. *N*., Inventor, B. R., Greiner, S.M., Mayer, K. M., Rivard, M. T., and Hamilton, R. J.	2012	USA	Discussion paper
Brawner, B.M., Volpe, E.M, Stewart, J.M., and Gomes, M.M	2013	USA	Qualitative research
Perlman, D.C., Gelpi-Acosta, C., Friedman, S.R., Jordan, A.E., and Hagan, H. 2015.			
USA	2015	USA	Qualitative research
Gassman, R.A., Agley, J., Fly, A.D., Beckmeyer, J.J., He, K., Sayegh, M.A.,and Tidd, D.	2016	USA	Qualitative research
Campbell, M.M., Susser, E., Mall, S., Mqulwana, S.G., Mndini, M.M., Ntola, O.A., Nagdee, M., Zingela, Z., van Wyk, S., and Stein, D.J.	2017	South Africa	Quantitative research

Further evaluation of the reports revealed that most of the papers were published in USA (*n*=9). In the UK, Canada, Norway, and South Africa, there was one paper published in each. Most of the papers were published between 2000 and 2010 (*n*=8), and five reports after 2010.

Hopia et al. (2016)^44^ raised some concerns in their review on the methodology of integrative reviews, especially on the issue of the methodological rigor followed and reported in publications. However, there are factors that may influence the execution of the study, for example, data itself, and therefore adaptations may be needed. In this study, in the phase of data search it became evident that only a few research reports on the topic existed. This observation strengthened the decision of integrative review as the methodological choice to enable inclusion of different types of data. There were several discussion papers written by experts in the area and included in the databases of peer-review publications. The small number of research reports made the evaluation of the quality of the original studies impossible, thus the descriptive details of the publications were reported instead in this review. The characteristics of the data also affected the decision to exclude gray literature from this review, although recommended by Whittemore and Knafl (2005),^39^ as the data itself already included texts which could be described as descriptions information sharing and considerations. However, the limited number of studies as well as the characteristics of the data should be considered as limitations of this study.

The reliability of this review was emphasized by both authors’ active involvement in different stages of the process and the collaboration of the librarian in the data search. In the data extraction process, both authors screened the reports independently. Consensus was sought in each of the screening phase with discussions and joint decision on included reports, described in Figure 1 following the renewed PRISMA guideline.^41^

In the analysis phase, the constant comparison method was applied with coding, extracting, comparing, and categorizing the findings as themes occurred in the data.^39,46^ Five themes emerged in the data: complexity with the capacity to consent, mixed emotions towards participation, factors influencing the decision to participate, nurses’ informed consent process competence, and variations between the consent procedures. The following result section describes the content of these themes.

## Results

### Complexity with capacity to consent

Two problems may occur when assessing the capacity of an individual with mental health problems to give informed consent: there may be a need to protect the person at that moment, but at the same time this excludes the person from the potential benefits of the study. Mental health disorders do not automatically mean reduced capacity to consent and the capacity to consent is not static.^47,48^ From the participant’s perspective, participation may also be a positive experience and therefore it should also be provided as a possibility to persons with mental health problems, instead of automatically excluding them from studies.^34^

In the context of genomic studies among persons with mental health disorders, recruiters but also the research ethics committees need to have expertise in the evaluation of the competence to consent, besides expertise in genetics. There is also a demand for continuous education and advice for research ethics committees to ensure they owe the latest knowledge available on these issues.^48^ Campbell et al.^49^ suggest the use of standardized informed consent screening tools to promote the quality of consent, while at the same time reminding that capacity to consent may not be affected by mental health problems alone but also other factors like poverty or challenges to access education and health care services.

### Mixed emotions towards participation

Brawner et al. (2013),^50^ Perlman et al. (2015),^51^ and Gasmann et al. (2016)^52^ describe some similarities between concerns, although in different contexts and with different participants. The concerns about confidentiality and discrimination were found to be potential factors to prevent participation, but there may also be more concrete issues, such as concern about the pain of a needle prick.^52^ In Perlman et al. (2014)^51^ study among drug users, concerns arose about the use of the results by law enforcement because of illicit drug use. Similarly concerns and mistrust were described about racism, especially with race-based genetic testing and the use of race or ethnicity as a predictor of genetic differences. Participants’ mistrust was also seen in genetic testing for the treatment and prevention of drug misuse; many disagree with the biological model on drug addiction.^51^ In the study of Brawner et al. (2013)^50^ some of the participants also mentioned ethnicity as a factor decreasing the trust they have in the research.

Similar concerns were reported in studies with adolescents. Gassman et al. (2016)^52^ described uncertainty about the idea of urine samples as they thought the results would be connected to their survey answers and be used for punitive purposes. Similarly, in Brawner et al.^50^ study the female adolescents described hesitation towards participation feeling that the laboratory findings were taken for testing, for example, pregnancy, illicit drug use or sexually transmitted infections and this information could be shared with their parents or guardians. Suspicious thoughts towards the use of DNA for different non-ethical purposes were also described.^50^

Brawner et al.^50^ found majority of the participants to have positive attitudes towards research, but also concerns about the information they receive and what takes place during the research. Participants were concern about transparency and privacy^50,52^ and therefore their suggested the blinding of the results from the research team, parents, or officials, like police.^50^ Participants needed to feel comfortable with the research or otherwise they would decline. Gassman et al. (2016)^52^ highlighted this in their study with youth, considering the possible need for a bigger role for parents in the study settings as they might want to get detailed information on the research procedures.

### Factors influencing the decision to participate

Already in the recruitment phase of a genetic study, there are factors that may be crucial when a potential participant is considering participation. Even the type of the biological sample gathered can make a difference,^50,52^ in which case, for example, “pep talks” by the research team and social support were described as important factors to support participation.^50^

Perlman et al. (2014)^51^ described some solutions to decrease potential participants’ concerns about their study with drug users. The setting and the professional, in this case a medical setting and a medical provider who asked for the informed consent, were found important, as well as the clear rationale for the study. In the study with the youth, the setting was important, as the school setting was not seen appropriate for medical procedures.^52^

The participants’ rationales in favor of testing were genetic testing with the purpose to decide between treatment options, or to learn how to provide the best possible help or prevent illness of the potential participant’s relatives.^40^ Interest in learning more on themselves, their health and their family may also motivate to participate.^50^

Besides these factors, speaking with peers who had been tested was found to be important to increase trust.^51^ The importance of their mother’s or friends’ support, or participation with peers was emphasized by female adolescents participating in Brawner et al. (2013) study.^50^ Social networks, such as social media and information spread by word of mouth, played an important role in increasing the interest in participation.^50^

Provision of incentives or compensation and their amount in proportion to uncomfortableness of the study may be motivational factors, as well as added participant autonomy, especially in studies with the youth and their parents.^50,52^ Autonomy can be increased with detailed, thorough information provision beyond standard consent information. This could be reached with specific guides besides the informed consent form.^50^

The competence of the researcher has been described by Gassman et al. (2016)^52^ as an ability to balance between participants’ requests and methodological precision. In the study by Campbell et al. (2017),^49^ the individual characteristics of the recruiters, such as their ability to improvise and simplify the explanations, were the most important predictors affecting the participants’ understanding of the study. Therefore, it is important that the recruiter understands the study well and is capable of clearly explaining it to the participants.^49^

In the informed consent process, there is a risk that the person obtaining the consent may themselves influence the situation.^53^ Confidentiality was described in several studies as one of the participants’ major concerns,^50–52^ which emphasizes the importance of a confidential and trusting relationship between the recruited and the potential participants. Cedar (2006)^53^ considers this especially in the situation where there may be a need to contact the genetic donor again, sometimes after a long time, and whether this contact should be made or not.^53^

### Nurses’ informed consent process competence

Nurses need to have up-to-date *knowledge* to be able to answer patients’ questions and to provide patient education on genomic testing and test results.^54,55^ This knowledge enables nurses to provide accurate information, but it also includes a patient’s right not to know. Therefore, nurses need knowledge of human genetics as well as ethical practices.^56^ In the field of stem cell studies, ethical practice furthermore demands knowledge of biological processes and the possible clinical uses.^53^ Similarly, Prows and Saldana (2009)^57^ described specific key points nurses need to understand about pharmacogenetic testing when working with children with mental health disorders. Nurses must be able to educate patients and families on these matters, in order to ensure they can provide this information later themselves, for example, if medication is selected and prescribed by another doctor or advanced practice nurse. As the knowledge of pharmacogenetic factors increases, nurses working with children and adolescents having mental health disorders need to familiarize themselves more and more with these issues.^57^

Nurses need to have *skills* to ensure that the patient gives an informed consent.^54,55^ According to Williams and Schutte (2000)^56^ nurses’ role as patient advocates is one of the crucial ones when working with persons seeking, managing, or coping with genetic information. This also includes support by nurses to help persons to cope with their personal genetic information about mental health conditions. From the heredity and family viewpoint nurses must have skills to obtain the family history and include this information into care planning.^56^ Besides the role of an advocate in the research process,^54^ nurses should provide the best possible care for patients at the same time.^30^

Within the context of stem cell studies, Cedar (2006)^53^ emphasizes nurses’ ability to describe these procedures clearly to patients to gain their informed consent. The skills to provide clear and detailed information may affect the very willingness to participate if misunderstandings and concerns can be decreased.^50,52^ This involves information on the research process but also on how the results will be used.^50^

In stem cell studies, nurses are often in ethically and morally challenging situations, and sensitivity is required as many of these matters are personal and private.^53^ A supportive *attitude* is also needed when the results are negative as Williams and Schutte (2000)^56^ described in their study on genetic testing with Huntington disease. Emotional distress, like sadness, worrying, and survivor guilt may be strong in these situations. In general, emotional support may be needed especially in the process of giving consent to genomic studies on mental health as the risk of stigmatization is high. Therefore, the provided support as well as education should also include family members in this process.^30,56^

### Variations between consent procedures

The complexity of informed consent to genomic studies is evident when considering the use of samples not only in the primary study but also in the possible secondary studies. Therefore, the broadness of consent needs to be carefully considered from different viewpoints. Contacting research participants may be difficult and ethically problematic as these contacts can be considered as intrusive. One option could be time-defined consent with description of possible secondary uses, which in turn should concern the same field of study as the primary one. A genetic study on depression as the primary study and the potential secondary study on other mood disorders in certain time limits, for example.^47^

Another approach to informed consent is described by Hem et al. (2006).^45^ Their observational study in the psychiatric ward environment described the challenges and solutions of informed consent in these circumstances. The process with informed consent was challenging; while the public supervisory body instructed otherwise, several experts on research and professional ethics, psychiatry and methodology supported continuance of the study. In this way already the continuity of the study was encouraged by several experts. The informed consent in this study was continually negotiated. In this negotiating process, the competence of the researcher was crucial: there was a need to have competence not only in research and methodology but also in the therapeutic practices based on clinical experience in psychiatry. This ensured ethically high standard proceeding.^34^ Bevan et al. (2012)^30^ also referred to Hem et al.’s study^45^ and they consider this approach of a continuous process potentially beneficial not only in qualitative, but also in quantitative designs of genomic studies.

DeCamp and Sugarman (2004)^47^ highlighted two approaches towards informed consent if person has a decreased capacity to give informed consent. There could be a modification of the traditional informed consent process, including, for example, educational interventions for these persons (see also Campbell et al. 2017)^49^ or proxy consent from a legally authorized person, often by a family member. However, the latter faces potential issues as family members may not always follow the interests of the person they represent, and therefore the first option might be preferable.^47^

With adolescents as potential participants some specific issues should be acknowledged, such as the role of parents or guardians in the consent process. This includes consideration of how much and what information to share with them as adolescents may not want to share their private or sensitive matters.^50^ However, families as well as others in the social network of adolescents, such as the school personnel, can also give valuable recommendations for the procedures already in the planning phase of the research and therefore their involvement should be carefully considered.^52^

In the clinical genomic trials one of the ethical issues is the consent form itself, which should reflect the trial and not the clinical treatment.^53^ The form should be clear enough to enable the decision-making of the patient and possible legal representative.^30,48^ Besides the form this decision-making process can be supported by guidelines and other education materials on genetic studies.^48,50,51^

## Discussion

Findings from this study provides an overall picture of informed consent practices in the context of genetic studies on mental health from the nurse’s viewpoint. There are various factors which may affect both the willingness to participate in the study and the informed consent process. These aspects should be carefully taken into account already in the planning phase of research. At the same time this study provides information on the research gaps in this area. These are described below together with implications to practice, education and policies.

### Implications for practice

Mental health problems can affect a person’s capacity to give their informed consent, but this does not automatically apply to all.^47,48^ According to Sundby et al. (2019)^35^ stakeholders who were involved in genomic research in mental health wanted to be more actively involved and considered consent as a reciprocal transaction between the participants and the researchers in the project. In addition, more active collaboration between researchers and clinical geneticists was desired. This follows the idea of recovery approach with true collaboration and inclusion.^16–18^

Potential participation raised several mixed emotions and therefore the activities towards decreasing the concerns are highly important. It was found that majority of the participants had positive attitudes towards research, but also concerns about the information they receive and what happens to them in the research,^50^ especially in the context of transparency and privacy.^50,52^ Concerns on confidentiality and discrimination were found to be potential factors to prevent participation,^52^ especially if potential participants have doubts on race-based genetic testing and about the use of race or ethnicity as a predictor of genetic differences.^50,51^ Issues that remain unclear cause suspicion and thus also undermine faith in research. In addition, the decreased willingness to participate may also affect the reliability of research results. As Brawner et al. (2013)^50^ argued autonomy may be increased with detailed, thorough information giving beyond standard consent information as well as with specific guides besides the informed consent form.

The secondary use of samples, which is common in genomic studies, may cause uncertainty among participants. The process of contacting research participants in an ethically and legally acceptable manner may be challenging.^47^ It should be noted that legislation also guides the informed consent process very strictly, for example, data protection legislation protects the rights of research participants.^58^

Several factors affecting the willingness to participate should be recognized and acknowledged already in the planning of the research (e.g., Reference 50). One is a characteristic of genetic research: the individual’s consent also affects family members. As Brawner et al. (2013)^50^ found out, potential participants’ interest in themselves and family could also positively affect participation as well as interest in their health. Support from their friends, peers or mother were important factors in affecting female adolescents’ participation.^50^ However, it should be noted that relationships within the family may not always be easy. As Cowley (2016)^59^ discovered, people who participate in genetic testing usually express their decision to be in line with “common sense,” whereas those who decided not to participate are considered absurd. Participants might spoke negatively of family members who refused testing, although there is always a possibility to not to participate.^60,61^ These discussions showed that genetic testing was seen both as an independent choice and as a responsibility. The right to be unaware seemed to be an important moral structure that helped to ethically manage the unpopular decisions made by family members. In the light of Cowley’s (2016)^59^ study, the dismantling of the “right not to know” by genomics can have subtle but profound consequences for family relationships.

Overall, the reasons for participating in a study are very understandable, such as decisions between treatment options, learning about how to help best or prevention of a relative’s illness.^51^ However, it should be noted that recommendations from others may be a major factor when deciding to participate or not to the study. For example, speaking with peers who have been tested was found to be important in increasing trust.^51^ Social networks such as social media and word of mouth, peers talking together, were also described as important in increasing the interest in participation.^50^ As Cowley (2016)^59^ pointed out, there may be an opportunity to pressure someone to participate in the study on behalf of family members. The recruiter should take this into account and emphasize autonomy and voluntariness.

The form of informed consent could also decrease the willingness to participate. It has been argued that there may be problems with “open” informed consent as some of the studies show uncertainty about genetic testing from the viewpoint of discrimination, cloning or possible false criminal implications; sometimes because of previous experiences on these issues with legal authorities.^50–52^ This is especially challenging in genomics and genetic studies in which future use might be unspecified.^61^

### Implications for education

In general, the findings of this study emphasize the need for more education [e.g., Reference 49] The Consensus Panel on Genetic (2006)^28^ has introduced genetic core competencies which all health care professionals should have. These include a set of knowledge, skills and attitudes, for example, basic human genetics terminology, family genetic history assessment and ethical, legal, and social implications related to genomics. This study shows that in mental health there is a need for recruiter to have also therapeutic competence.^45,49^ These aforementioned competences demand a development both in the development of curricula as well as in the contents of lifelong learning.^26^

In health care, also in mental health settings, nurses are the largest professional group, and therefore nurses’ competence as recruiters, information providers, and patient and family advocates on the process of informed consent related to genetics and genomics is crucial.^49^ As nurses are often also the ones working closest with patients, it could be easy for patients to discuss about participation and other research issues with nurses.^26^ This demands nurses to have up-to-date knowledge to be able to answer patients’ questions also on genomic testing and test results^54,55^ as well as to promote patients’ autonomy and the right to not know their test results.^56^

Therefore, it could be stated that the ability of nurses, to support clients and patients across the care continuum in acquisition and use of genetic information is a critical prerequisite for the successful integration of genetic information into the delivery of comprehensive mental health care services.^45^ Campbell et al. (2017)^38^ also pointed out that individual characteristics of the recruiters, in this case nurses, were the most important predictors of the participants’ understanding of the study. This demands that nurse him/herself understands the study well and is capable to clearly explain it to the potential participant. This might be a challenge if only some genomic knowledge is included in nurses’ curricula.^20^

Ethical sensitivity is needed in the informed consent practices. The findings of this study highlighted that increased complexity in the health care practice has led to higher demands on staff to handle ethically sensitive issues.^49^ Ethical sensitivity plays a central role especially in genomics due to stigmatizing conditions, like mental health problems. Ethical sensitivity includes the sensitivity to identify difficult ethical issues^50^ and requirements to obtain knowledge of human genetics as well as ethical practices,^56^ in order to discuss difficult issues related to genetics and genomics. In addition to this, in stem cell studies ethical practices also demand nurses to have knowledge of biological processes and the possible clinical uses.^53^ The education of nurses has not kept pace with this development. In particular, the pharmacogenetic competence of nurses should be strengthened in the care of mental health disorders.^57^

It has been noticed that educators’ personal and professional ethos is crucial to student learning, personal growth, and ethical reasoning. Therefore, it is important to further develop not only the nurses but also educators’ training regarding ethical competence,^64^ especially in the field of genomics^65^ also in mental health.

### Implications for research

Torracco (2016)^40^ described two types of topics in an integrative review: mature and new topics. This review showed that the topic itself has been mostly studied or discussed during the first decade of 2000 and since then there has only been some individual reports. This may reflect the development of genetic studies from the viewpoint of mental health nursing, but the number of reports does not support the idea of a mature topic with a wide literature base.^40^

In total the number of papers on this topic was very low, especially with research-based articles. This could be considered as the most significant limitation of this study and at the same time it indicates a gap of research on this area. A need for updated research-based knowledge, research with different methodologies and different perspectives to cover the important viewpoints of informed consent processes in mental health settings remains. Especially the voice of those involved would be important to hear and therefore more research is needed from the viewpoint of both the potential or actual participants, the persons with lived experience on mental health challenges, as well as the nurses working in the field of genetic studies on mental health. Follow-up studies, for example, on concerns with open informed consent, would be already possible, but none were found during the search phase. With more high-quality research on the topic, we can develop the practices as well as education to ensure realization of the recovery approach and true further collaboration in the context of genetic studies on mental health.

### Implications for policies

The findings of this study reveal different themes on informed consent practices with genetic studies on mental health. However, the discussion concerning the right to not to consent and to not to participate was very limited in the original articles despite its importance from the viewpoint of self-determination^60^ Exceptions on this are very seldom even in intervention studies^66^ and therefore it would be important to highlight this possibility as a true choice in research policies as well as in education.

The recovery approach is the main guiding approach today in different mental health settings in several countries,^16^ but still there is a need to develop more collaborative practices. In this study, it was shown that the discussions with peers were found as a significant factor affecting the considerations on participation of the genetic study on mental health. This is important finding from the recovery viewpoint as it encourages to collaborate more with persons with lived experiences on mental health also in this field of research.^67–69^

## Conclusion

This study reveals the scarcity of studies on informed consent practices in the context of genomic studies on mental health. There is a need for more updated international research on this topic in the context of different international and national guidelines, legislation, and directives when biobank activities and the number of genetic studies has increased.

A need for more research remains from the nurses’ viewpoint as well. This review shows some factors which might affect the informed consent processes, but more research-based knowledge is needed. It is part of the nurses’ competence to encounter the potential participants; to share knowledge, understanding and help to patients and families with experiences on mental health problems, and as part of their professional ethical conduct to ensure that participants are met as active subjects with the option to decide if they want to participate in the study or not. This study provided a viewpoint to the more collaborative research activities with people with lived experiences also in this field of research following the ideas of recovery approach.
